# Cortical connective field estimates from resting state fMRI activity

**DOI:** 10.3389/fnins.2014.00339

**Published:** 2014-10-31

**Authors:** Nicolás Gravel, Ben Harvey, Barbara Nordhjem, Koen V. Haak, Serge O. Dumoulin, Remco Renken, Branislava Ćurčić-Blake, Frans W. Cornelissen

**Affiliations:** ^1^Laboratory of Experimental Ophthalmology, University Medical Center Groningen, University of GroningenGroningen, Netherlands; ^2^Laboratorio de Circuitos Neuronales, Centro Interdisciplinario de Neurociencia, Pontificia Universidad Católica de ChileSantiago, Chile; ^3^Experimental Psychology, Helmholtz Institute, Utrecht UniversityUtrecht, Netherlands; ^4^Donders Institute for Brain, Cognition and Behaviour, Radboud UniversityNijmegen, Netherlands; ^5^NeuroImaging Center, University Medical Center Groningen, University of GroningenNetherlands

**Keywords:** RS-fMRI, population receptive fields, connective field modeling, connectivity mapping, visuotopic maps

## Abstract

One way to study connectivity in visual cortical areas is by examining spontaneous neural activity. In the absence of visual input, such activity remains shaped by the underlying neural architecture and, presumably, may still reflect visuotopic organization. Here, we applied population connective field (CF) modeling to estimate the spatial profile of functional connectivity in the early visual cortex during resting state functional magnetic resonance imaging (RS-fMRI). This model-based analysis estimates the spatial integration between blood-oxygen level dependent (BOLD) signals in distinct cortical visual field maps using fMRI. Just as population receptive field (pRF) mapping predicts the collective neural activity in a voxel as a function of response selectivity to stimulus position in visual space, CF modeling predicts the activity of voxels in one visual area as a function of the aggregate activity in voxels in another visual area. In combination with pRF mapping, CF locations on the cortical surface can be interpreted in visual space, thus enabling reconstruction of visuotopic maps from resting state data. We demonstrate that V1 ➤ V2 and V1 ➤ V3 CF maps estimated from resting state fMRI data show visuotopic organization. Therefore, we conclude that—despite some variability in CF estimates between RS scans—neural properties such as CF maps and CF size can be derived from resting state data.

## Introduction

The human visual cortex is a highly complex and interconnected system operating at various temporal and spatial scales, and as such, non-invasive assessment of the neural correlates of human visual processing are of great importance. A significant contribution toward understanding human visual processing can be made by studying cortico-cortical interactions between different visual areas (Heinzle et al., 2011; Haak et al., 2013; Raemaekers et al., 2013). One way to study these neural correlates is by examining spontaneous blood-oxygen level dependent (BOLD) co-fluctuations during resting state (Heinzle et al., 2011; Raemaekers et al., 2013). Given that resting state BOLD fluctuations are partly shaped by the underlying functional and neuroanatomical organization (Biswal et al., 1997; Logothetis, 1998; Raichle et al., 2001; Boly et al., 2007; Deco et al., 2011; Hutchison et al., 2013a; Wang et al., 2013), analysis of resting state activity offers a possibility to examine intrinsic functional connectivity of the visual system as well as the extent of variability of these processes.

Although functional magnetic resonance imaging (fMRI) indirectly measures neural activity, accurate methods to map neural response selectivity in the early visual cortex from the BOLD signal have been developed (Engel et al., 1997; Smith et al., 2001; Dumoulin and Wandell, 2008). With these methods, the unifying concept of classical receptive field (Hubel and Wiesel, 1962) has found its place in fMRI, under the definition of population receptive field (pRF). The term pRF was first used to describe population encoding in macaque early visual areas (Victor et al., 1994). Used in fMRI, the term describes the aggregate responses of fMRI recording sites (voxels) to presented stimuli, in terms of the position and size of the visual field area to which each recording site responds.

The parametric modeling approach of the pRF techinque has allowed non-invasive investigation of neural response selectivity, its cortical organization, and the computational properties of the visual system. A recent complementary method, called connective field (CF) modeling (Haak et al., 2013), extends this type of analysis to model cortico-cortical interactions in terms of spatially localized patterns of functional connectivity. Specifically, this method enables characterization of a recording site in terms of aggregate cortical activity in another brain area, thus extending the concept of receptive field from a description of preferred locations in visual (stimulus) space to preferred locations on the cortical surface.

CF modeling was originally conceived as a method to analyze responses evoked by visual field mapping (VFM) stimuli, though the analysis does not use a description of the stimulus. As such, it could in principle be applied to explore cortico-cortical connectivity profiles during different experimental conditions as well as resting state. To realize this potential, a number of questions must be addressed. In this paper, we try to provide answers to at least four of them. First, how do we measure CF models in the presence of substantial physiological measurement noise? Second, how much scan time is sufficient to achieve accurate discrimination of CF models obtained from resting state data? Third, how do CF parameters obtained from resting state compare to those obtained from stimulus-evoked activity? Four, to what extent do CF parameters vary between resting state scans?

While previous studies have examined cortico-cortical interactions in the early visual cortex during resting state (Heinzle et al., 2011; Raemaekers et al., 2013), our current study focuses on the application of the CF method. These previous studies used model-free approaches whereas the CF method is a model-based approach. To the extent that the model adequately describes the underlying neuronal activity, model-based approaches provide summary descriptions of aggregate neural activity, which is another reason to examine the application of the CF method to analyze resting state fMRI data.

## Materials and methods

### Participants

We recruited four subjects with normal visual acuity (age: S1 = 26, S2 = 30, S3 = 31, S4 = 40 years old). Experimental procedures were approved by the medical ethics committee of the University Medical Center Utrecht.

### Stimulus

Visual stimuli were presented by back-projection onto a 15.0 × 7.9 cm gamma-corrected screen inside the MRI bore. The subject viewed the display through prisms and mirrors, and the total distance from the subject's eyes (in the scanner) to the display screen was 36 cm. Visible display resolution was 1024 × 538 pixels. The stimuli were generated in Matlab (Mathworks, Natick, MA, USA) using the PsychToolbox (Brainard, 1997; Pelli, 1997). The mapping paradigm consisted of drifting bar apertures at various orientations, which exposed a 100% contrast checkerboard moving parallel to the bar orientation. After each horizontal or vertical bar orientation pass, 30 s of mean-luminance stimulus were displayed. Subjects fixated a dot in the center of the visual stimulus. The dot changed colors between red and green at random intervals. To ensure attention was maintained, subjects pressed a button on a response box every time the color changed (detailed procedures can be found in Dumoulin and Wandell, 2008; Harvey and Dumoulin, 2011). The radius of the stimulation area covered 6.25° of visual angle from the fixation point.

### Resting state

During the resting state scans, the stimulus was replaced with a black screen and subjects closed their eyes. We chose this so that there was no visual input; neither from outside the stimulus area (hence eyes closed) nor from light coming through the eyelids (hence the black screen). The lights in the scanning room were off and blackout blinds removed light from outside the room. The room was in complete darkness.

### Data acquisition

Functional T2^*^-weighted 2D echo planar images were acquired on a 7 Tesla scanner (Philips, Best, Netherlands) using a 32 channel head coil at a voxel resolution of 1.98 × 1.98 × 2.00 mm, with a field of view of 190 × 190 × 50 mm. TR was 1500 ms, TE was 25 ms, and flip angle was 80°. The volume orientation differs between subjects, though in all cases it was approximately perpendicular to the calcarine sulcus. High resolution T1-weighted structural images acquired at 7T using a 32 channel head coil at a resolution of 0.49 × 0.49 × 0.80 mm, with a field of view of 252 × 252 × 190 mm. TR was 7 ms, TE was 2.84 ms, and flip angle was 8°. We compensated for intensity gradients across the image using an MP2RAGE sequence, dividing the T1 by a co-acquired proton density scan of the same resolution, with a TR of 5.8 ms, TE was 2.84 ms, and flip angle was 1°. In total, eight 240-volumes functional scans were acquired; comprising 5 resting state scans (RS) and 3 interleaved VFM scans. The first scan was a RS scan. Physiological data were not collected.

### Preprocessing

First, the T1-weighted structural volumes were resampled to 1 mm isotropic voxel resolution. Gray and white matter were automatically segmented using Freesurfer and hand edited in ITKGray to minimize segmentation errors (Teo et al., 1997). The cortical surface was reconstructed at the white/gray matter boundary and rendered as a smoothed 3D mesh (Wandell et al., 2007). Motion correction within and between scans was applied for the VFM and the RS scans (Nestares and Heeger, 2000). To clean the resting scan signals from DC baseline drift and reduce high frequency nuisance from physiological variation, time courses were band pass filtered with a high-pass discrete cosine transform filter (DCT) with cut-off frequency of 0.01 Hz and a low-pass 4th order Butterworth filter with cutoff frequency of 0.1 Hz. Finally, functional data were aligned to the anatomical scans (Nestares and Heeger, 2000) and interpolated to the anatomical segmentation space.

### Analysis

#### Population receptive field mapping

Early visual areas V1, V2, and V3 were mapped using the pRF method (Dumoulin and Wandell, 2008). The method uses a parameterized forward model of the underlying neuronal population, a description of the hemodynamic response (HRF), and the stimulus aperture. The model we chose corresponds to a circular Gaussian characterized by three parameters: x and y (positions), and size (σ). A set of candidate pRF models are combined with the stimulus aperture to generate predictions of the neural responses each candidate pRF would produce. Subsequent convolution of this predicted neural response time course with the HRF give a set of candidate predicted fMRI response time courses for each combination of pRF parameters. The best fitting predicted fMRI time courses and their associated pRF parameters are then chosen to summarize the response of each recording site (Dumoulin and Wandell, 2008).

#### Connective field mapping

CF model parameters were estimated for both the VFM and RS scans using the CF modeling method described by Haak et al. (2013). CF models summarize the activity of each recording site in a target region of interest (ROI) in terms of the aggregate activity contributed by a set of recording sites in a source ROI (Haak et al., 2013). Specifically, the BOLD activity over a particular part of a source region (the CF) is integrated (summed) to yield the BOLD activity at a target recording site, whose neural response we are trying to describe. As we aim to determine the source CF for all target recording sites within an ROI simultaneously, we describe a target visual field map ROI (i.e., V2 or V3). As candidate source CFs are limited to a particular visual field map, this is described as the source ROI (here, always V1). First, a discrete parameter space of 2-dimensional Gaussians of different candidate sizes (σ) is generated for each candidate location (each recording site inside the source ROI, V1), giving a set of candidate V1-referred CF models. In the next step, similarly to the pRF approach, a candidate predicted time course is generated for each candidate CF model by calculating the Gaussian weighted sum of the measured signals from the candidate CF (including the preferred recording site and its neighbors). These candidate time courses predictions are compared to the measured time course of each recording site in the target ROI (V2 and V3), and the best fitting prediction and its associate V1-referred CF parameters are chosen for each target recording site. Furthermore, because CF preferred locations in V1 cortical surface are associated with preferred visual field positions during pRF mapping, coordinates in visual space can be inferred for target recording sites. This allows the reconstruction of visuotopic maps even in the absence of stimuli. Note that the size of a CF represents the Gaussian spread along the cortical surface (mm) and is defined as the shortest path distance between pairs of vertices in the 3D mesh associated with the gray/white matter border. The location and size of the ROIs are defined during pRF mapping. These parameters (location and size of the source ROI) may restrict CF position but not CF size. By emphasizing the spatial profile of functional connectivity, a CF allows to examine spatially localized connectivity patterns among brain areas. As with most functional connectivity measures, CF models do not infer the temporal order of the responses in target and source recording sites.

#### Discriminability criterion

By emphasizing local over long-range functional connectivity, biologically inspired models like pRF and CF are generally robust to global effects (i.e., physiological noise). Nevertheless, evaluation of model significance can be frustrated by the noisy and non-stationary nature of the time series obtained from resting state. To overcome this issue and assess the statistical significance of CF models estimated from the RS, we apply a strategy based in surrogate data testing.

First, we distinguish the contribution of topographically organized BOLD co-fluctuations from spatially uncorrelated random BOLD fluctuations. This distinction allows defining a criterion in terms of model discriminability. In this context, we define discriminability as the distinction between topographically organized BOLD co-fluctuations and spatially uncorrelated random BOLD fluctuations. To determine model discriminability, we estimated null distributions from the variance explained (VE) of CF models obtained from surrogate V1 BOLD time courses. To generate these surrogate BOLD signals, artificial time courses were produced with the iterative amplitude adjusted Fourier transform (iAAFT) method (Schreiber and Schmitz, 1996; Venema et al., 2006). This method randomizes the phase of the original signal, but preserves its autocorrelation, linear structure, and amplitude distribution. The spatial correlation between BOLD time courses in the source region is lost but their fundamental statistical properties are preserved. Each CF model estimation was accompanied of an estimation based on surrogate time courses. For the present analysis, the null distributions obtained from 240 volumes (each RS scan) are comparable across subjects and target ROIs (V2 and V3); therefore, we combined all estimates into one null distribution and used the 5th percentile as discrimination threshold.

Second, we estimated the amount of data that is sufficient to discriminate RS-based CF models by examining the dependence of discrimination accuracy on data quantity. First, CF models were calculated for different amounts of RS data (both for original and for surrogate data). Segments of 40, 80, 120, 160, 200, and 240 volumes starting from the beginning of each RS scan were used. Next, VE estimates (adjusted for the degrees of freedom in each amount of volumes) were grouped according to their corresponding segment length, obtaining original and null VE distributions for each amount of volumes. These distributions allow the application of a receiver-operator characteristic (ROC) analysis. By assessing the performance of a binary classifier as its discrimination threshold is varied, ROC analysis provides quantitative measures of model discrimination performance. To discriminate CF models attributed to genuine BOLD co-fluctuations from those attributed to random BOLD activity, the corresponding VE cutoff threshold is moved from 0 to 1 across the original and the null distributions, producing a contingency matrix of true positives (*hits*), false positives (*false alarms*), true negatives (*correct rejections*), and false negatives (*miss*). Using the contingency matrix, values of true positive rate (*sensitivity*) and false positive rate (*1-specificity*) are computed and plotted as ROC curves. In ROC space, a diagonal line corresponds to random discrimination. The area under the ROC curve (AUC) is commonly used to quantify classifier discriminability, with a value of 0.5 corresponding to random, and a value of 1 to perfect, classification. We choose informedness as our discriminability index, which corresponds to twice the area between the curve and the diagonal: 2^*^AUC-1 (Hanley and McNeil, 1982; Fawcet, 2006). It has the advantage that 0 represents random, and 1 represents perfect classification. Finally, we estimated the dependence of discrimination accuracy on the EV cutoff threshold by calculating the F_1_ score for each amount of volumes.

#### Spatial analysis

In the spatial domain, we estimate CF size change and position scatter during RS using VFM-based size and position as reference.

First, to assess CF position variability in the RS, we assume that CFs are topographically organized. This implies that neural activity in neighboring cortical locations in the target ROI may correlate with neural activity in neighboring cortical locations inside the source ROI that represent the same portions of visual space, as shown by VFM. This assumption allows us to estimate position variability as position scatter of V1-referred CFs by calculating their displacement on the V1 cortical surface with respect to their VFM-based reference positions.

We proceeded as follows: for each recording site in the target ROI, position scatter was calculated as the shortest distance along the cortical manifold between the VFM-based center position and the RS-based position. This distance was computed in millimeters using Dijkstra's algorithm (Dijkstra, 1959). Estimates whose associated models scored a VE above discrimination threshold (0.35 VE) were retained. To quantify the variability in position scatter for each subject and each RS scan, the median (to assess tendency) and the median absolute deviation (MAD; to assess dispersion) were calculated for each RS scan and subject. To assess RS scan-to-scan variability, we also calculated these values for all RS scan pairs. In order to determine a possible influence of cortical distance (i.e., shared vasculature, spatial blurring), we compared position scatter as a function of the distance between CF centers and their associated recording sites in the target area. We then compared position scatter as a function of VFM-based reference eccentricity. Finally, agreement in eccentricity estimates was quantified by calculating linear correlation coefficients for VFM- and RS-based eccentricities.

Second, we examined differences in size for V1 ➤ V2 and V1 ➤ V3 models between RS- and VFM-based estimates. RS-based size estimates for V1 ➤ V2 and V1 ➤ V3 from all participants were grouped by map combination and compared to those obtained based on VFM using a two-sample Kolmogorov–Smirnov test (KS-test). Subsequently, we examined the relation of RS-based CF size as a function of VFM-reference eccentricity by binning eccentricity in bins of 1° and calculating linear fits over the mean with bootstrapped confidence intervals (1000 iterations).

## Results

### Deriving connective field models based on resting state fMRI data

**Figure 1:**
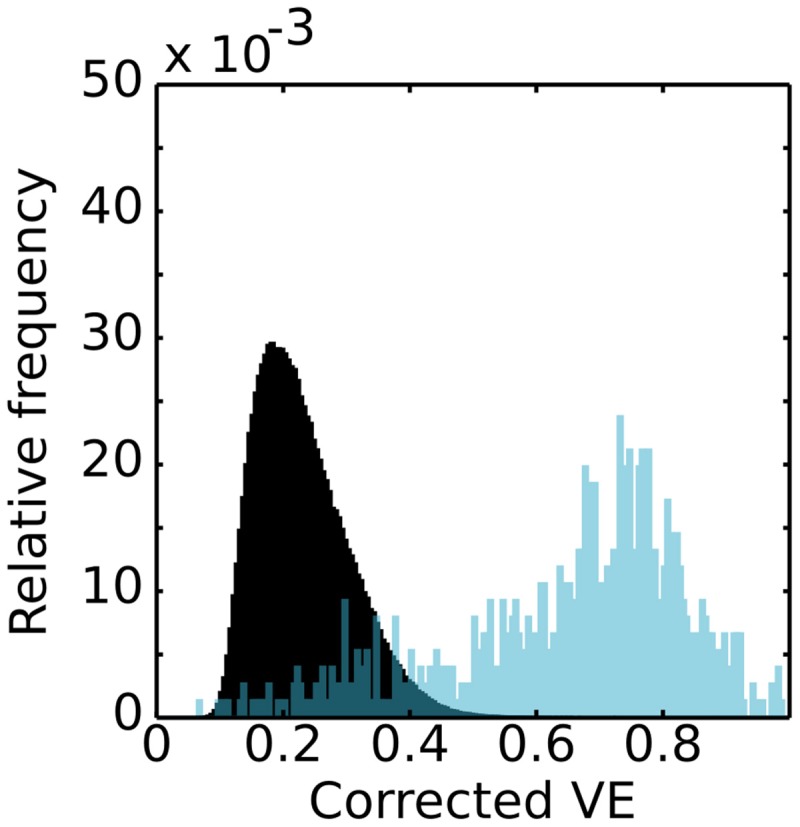
**Distributions of variance explained**. Histogram of relative frequency of recording sites in V1-referred connective field models in V2 as a function of their variance explained. The black distribution represents the hypothesis of non-discriminability (noisy and spatially uncorrelated signals obtained with the IAAFT method) and was generated by fitting connective field models to surrogate BOLD signals. The blue distribution illustrates a typical outcome for an actual resting state scan.

Our first analysis concerned two questions: whether CF models could be obtained in presence of substantial physiological measurement noise; and, if the models obtained could be discriminated based in the contribution of genuine spontaneous BOLD co-fluctuations. Figure 1 shows the distributions of VE for actual (*blue*) and surrogate (*black*) RS data. We used the VE of CFs obtained from surrogate RS data as null-distribution (240 volumes, TR: 1.5 s). The VE cutoff threshold was estimated based on the 5th percentile of the null-distributions and lies around ~0.35 VE for all subjects. The majority of the models have a VE that exceeds this cutoff threshold. Importantly, this analysis demonstrates that the estimation of CF models based in genuine spontaneous BOLD co-fluctuations is possible even in presence of substantial physiological measurement noise. Nevertheless, we cannot determine the effect that these confounds exerts in the estimation of CF parameters.

**Figure 2:**
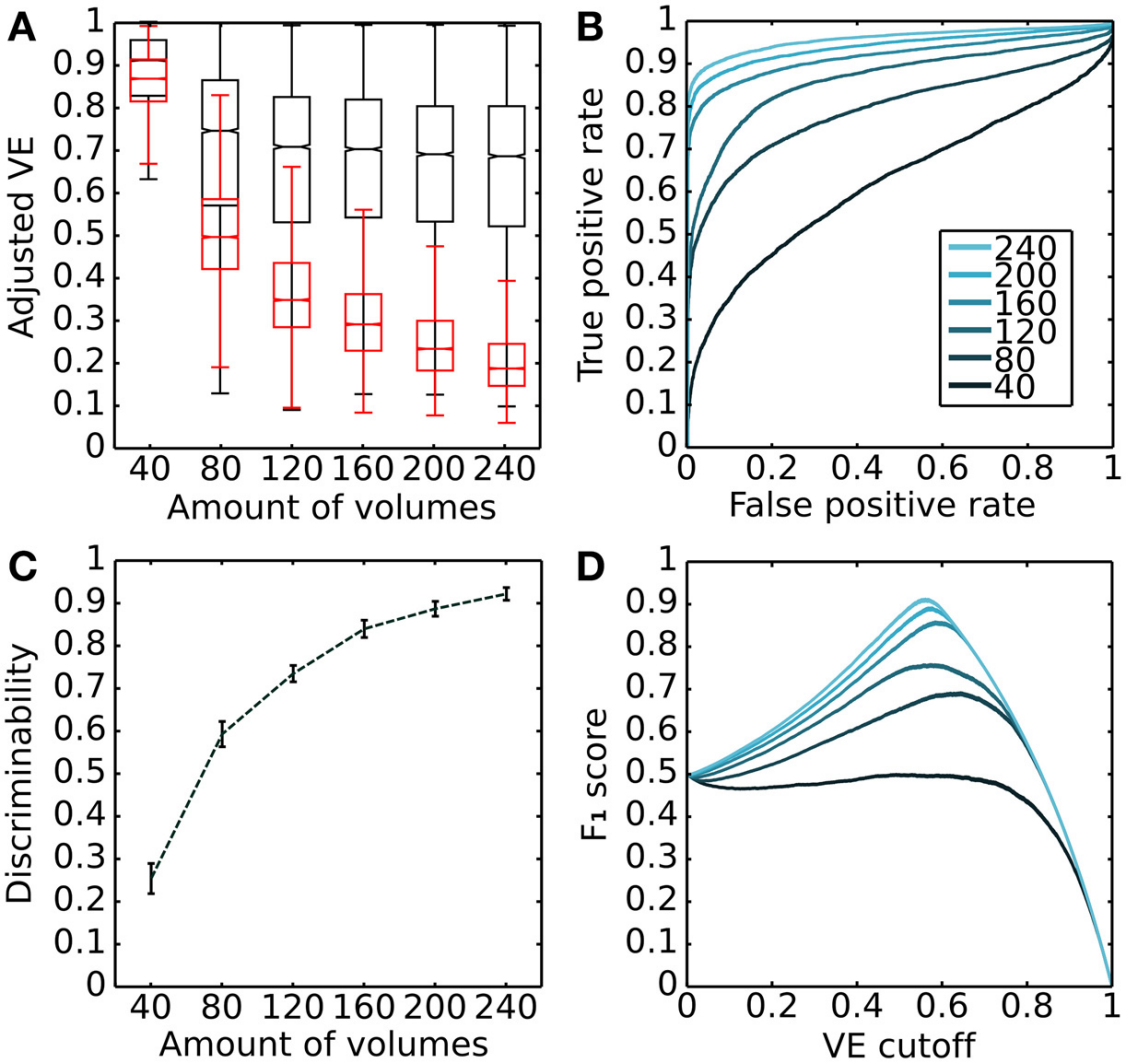
**Overall modeling performance characteristics for a single subject**. **(A)** Dependence of variance explained on the amount of volumes. The whisker box-plots illustrates the distributions of variance explained adjusted by degrees of freedom. Red distributions were obtained from RS data and black distributions from surrogate RS data. The central mark is the median, the edges of the box are the 25th and 75th percentiles, and the whiskers indicate the most extreme data points with an interquartile range of 1.5 (Tukey box-plot). **(B)** Receiver operating characteristic (ROC) curves corresponding to each amount of volumes. **(C)** Discriminability increases as a function of the amount of volumes (we choose discriminability index in the form of informedness: 2^*^AUC-1, equivalent to Gini coefficient). **(D)** Discrimination accuracy (*F*_1_ score) as a function of the adjusted EV cut-off threshold for each amount of volumes (colors as in **B**). Data are for V1 ➤ V2 CF models from subject 3.

In addition, we examined the dependence of discrimination accuracy on the amount of volumes included in the analysis. To do so, we calculated VE (adjusted for degrees of freedom) for actual and surrogate data for various amounts of volumes and applied a ROC analysis. Figure 2 summarizes the results of the analysis for a single subject (Subject 3). First, it shows the VE distributions for actual (*black*) and surrogate data (*red*) as a function of the amount of volumes included in the analysis. VE drops with the number of volumes, but drops more sharply for the surrogate data (Figure 2A). The resulting ROC curves are shown in Figure 2B; they show detection probability as a function of false alarm probability for each amount of volumes. Detection probability increases with the amount of volumes. Figure 2D shows discrimination accuracy (F_1_ score) as a function of the VE threshold for each amount of volumes analyzed.

This analysis also indicates that CF modeling could be based on even shorter scan periods with retaining reasonable discrimination accuracy. However, fewer models are expected to lie above threshold. Finally, it must be noted that, even though this analysis provides a strategy to optimize modeling accuracy by adjusting the VE cutoff threshold, in the remaining analysis we use a threshold of 0.35 VE, which corresponds to the 5th percentile of the null-distribution obtained after grouping the VE of surrogate RS-based models from all scans and subjects.

### Spatial aspects of resting state connective field map estimation

**Figure 3:**
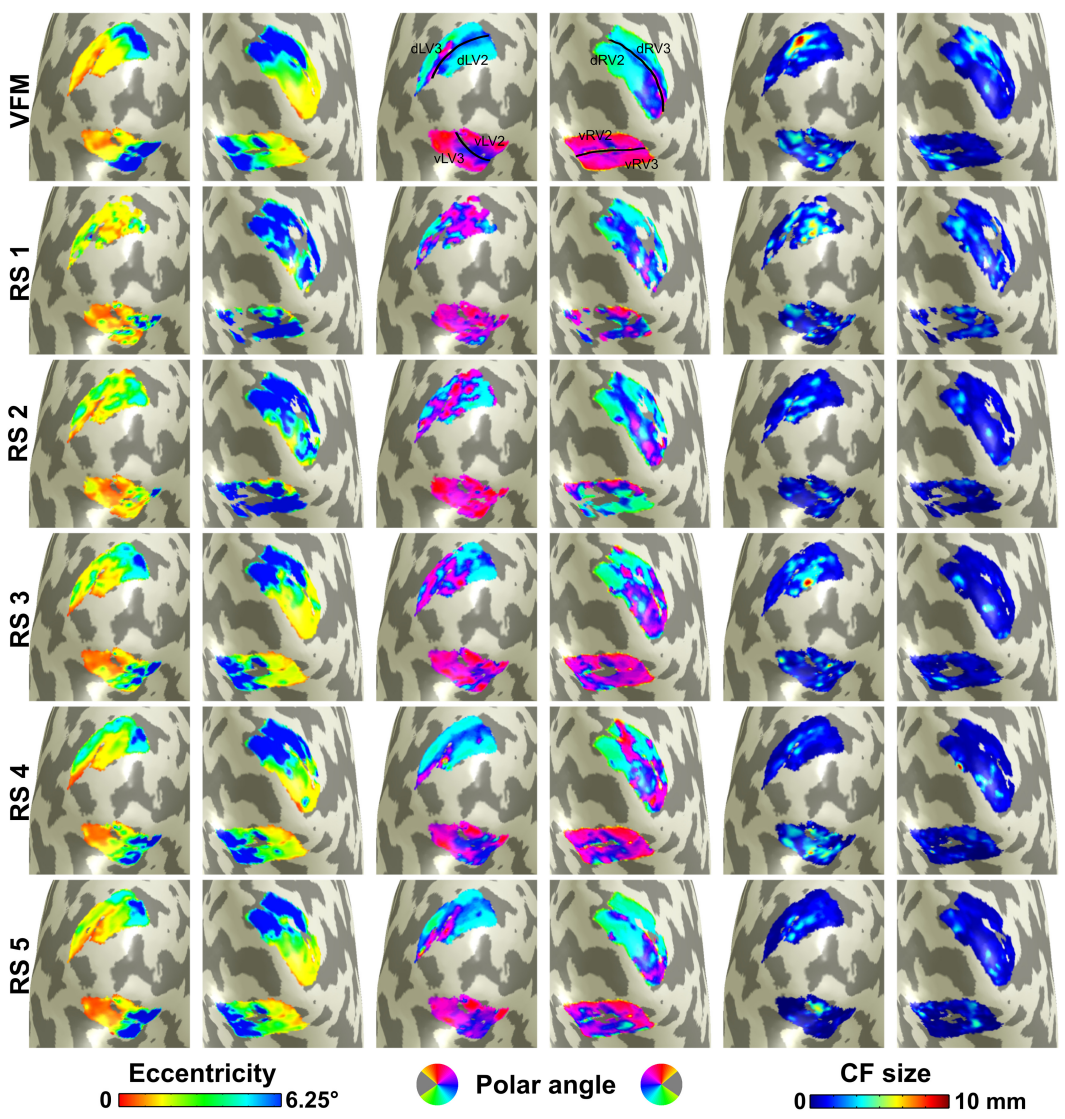
**Visualization of connective field maps for a single subject**. From left to right: eccentricity, polar angle, and size. **Top panel** corresponds to visual field mapping (VFM)-based estimates. **Lower panels** show parameter estimates for each resting state (RS) scan. For V1 ➤ V2 CF models, the position displacement in CF cortical location (in mm) between VFM- and RS-based estimates for RS1 to RS5 is: median (MAD) = 10.0 (5.4); 8.5 (5); 5.8 (3.7); 3.8 (3.4); and 4.1 (3.0), respectively [total = 5.4 (3.9)]. Corresponding position displacement values between RS4 and RS5 (the RS scans with lowest displacement: 4.1 (3.1); between RS1 and RS2 (the RS scans with highest displacement): 8.5 (5.8); between RS1 and RS4: 10.5 (6.6); when grouping results for all RS scan pairs: 8.6 (5.9). For V1 ➤ V3 CF models, the corresponding values are: 13.6 (6.3); 14.4 (6.8); 7.9 (5.4); 6.7 (5.5); and 7.1 (4.2) [total = 8.7 (5.5)]. Eccentricity and polar angle are inferred from V1 pRF mapping (see Materials and Methods for details). Data are for V1 ➤ V2 and V1 ➤ V3 models estimated for subject 3 (data for other subjects included in Supplementary Materials). A threshold of 0.35 VE was applied. Median cortical displacements reflect the agreement between RS and VFM maps and between different RS maps.

The next question we address is whether the topographical maps based on RS data have similar characteristics as the one based on VFM data (our current reference). Also, how variable are the results between RS scans? To provide an impression of this variability, Figure 3 shows both VFM and RS derived CF maps for a single participant (maps for other participants are shown in Supplementary Materials). V2 and V3 CF parameter maps (V1-referred) are plotted on a smoothed 3D mesh representing gray matter along the cortical surface. Eccentricity, polar angle and size (σ) are plotted in three columns. In top row of panels, CF parameters estimated based on VFM data are shown. These maps serve as our reference. In the lower rows of panels, these same parameters are plotted for all RS scans. As shown previously (Haak et al., 2013), the VFM derived maps show a clear retinotopic organization (note that in the context of CF modeling, eccentricity and polar angle maps are inferred from a pRF mapping and associated to each recording site in the source region, in this case V1). In some RS scans eccentricity and polar angles maps resembles the VFM-based reference, although some variability can be observed (Figure 3, RS4, RS5). To quantify the variability of the individual maps, the median position displacement in CF cortical location (relative to the VFM reference and between all RS scan pairs; in mm) and the MAD were calculated for RS1 to RS5 (values are reported in the legend of Figure 3). These values confirm the impression that RS4 and RS5 most clearly resemble the visuotopic organization observed in the VFM-based maps (results are shown for participant 3, those for the other participants are shown in the Supplementary Material).

**Figure 4:**
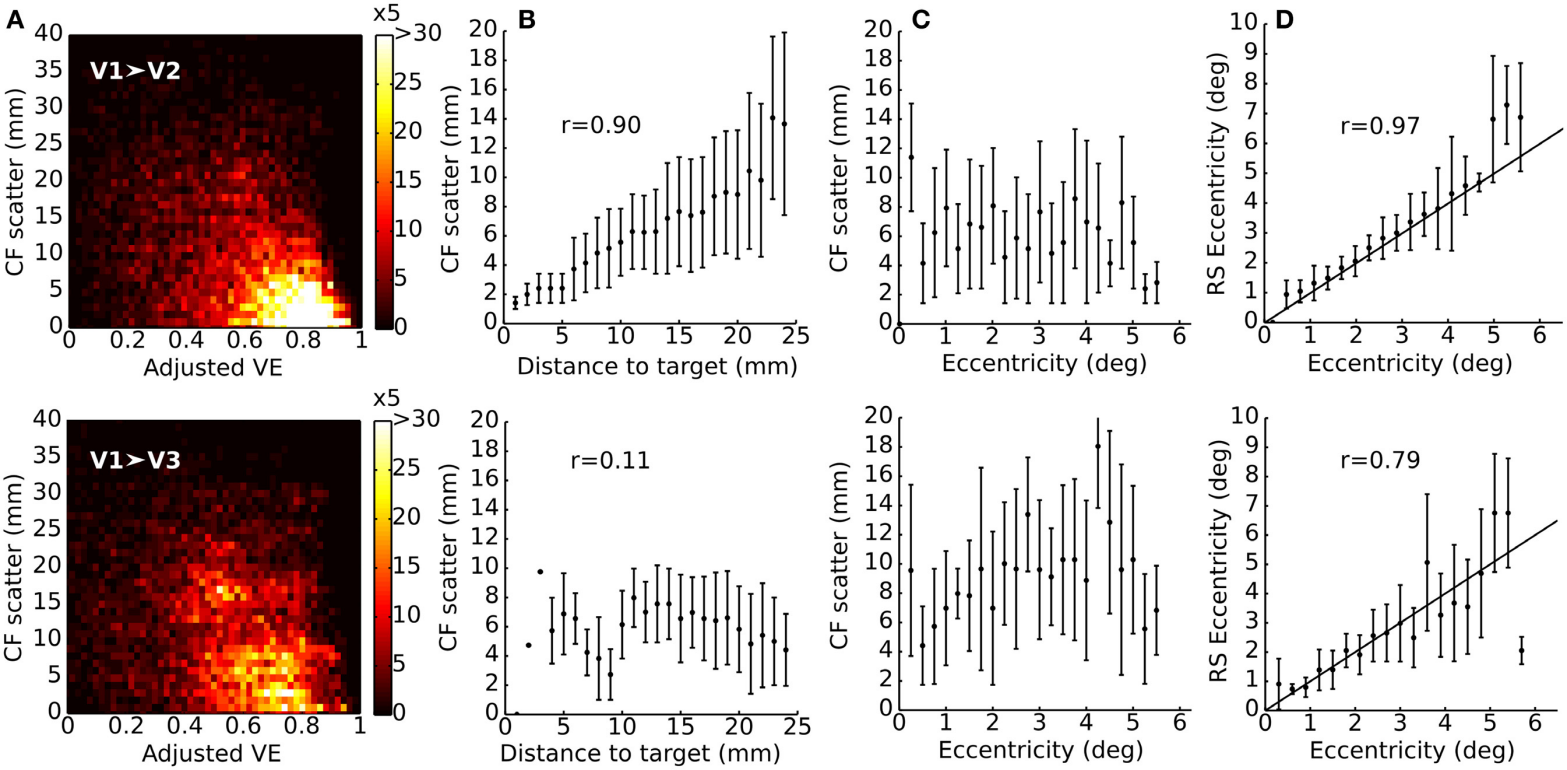
**Position scatter for V1-referred connective fields for a single subject. (A)** Joint histogram of cortical displacement in V1-referred CF centers as a function of adjusted VE. The goodness of fit tends to decrease with larger displacements (colorbar depicts frequency of voxels after grouping data from all RS scans; the number of voxels that entered the analysis is: 1622 for V1 ➤ V2 and 1467 for V1 ➤ V3). **(B)** Position scatter as a function of the distance from the target voxel. A cortical distance effect can be seen in V1 ➤ V2 (*R* = 0.90, *p* < 0.0001) but not in V1 ➤ V3 (*R* = 0.11, *p* < 0.0001). **(C)** No systematic deviations from the median distance are observed for eccentricity (data was binned in eccentricity bins of 0.25°). Points represent the median of each bin and error-bars the median absolute deviation for the corresponding bin. **(D)** There is good agreement between RS-based eccentricity and VFM reference eccentricity (V1 ➤ V2: *R* = 0.97, *p* < 0.0001; V1 ➤ V3: *R* = 0.70, *p* < 0.0001) (data was binned in eccentricity bins of 0.25°. Points represent the median of each bin and error-bars the median absolute deviation for the corresponding bin). Data are from subject 3. A cutoff threshold of 0.5 VE (*F*_1_ ~0.85) was applied in **(B–D)**.

Figure 4A plots the change in V1-referred CF center position between RS- and VFM-based reference position as a function of VE (of the RS model). CFs with higher VE show smaller cortical displacements. The majority of CFs (as indicated by the heat map) have a high VE and show relatively small displacements. Figure 4B shows a distance effect for V1 ➤ V2 (*R* = 0.90, *p* < 0.0001) but not for V1 ➤ V3 (*R* = 0.11, *p* < 0.0001). Figure 4C shows that there are no systematic deviations from the median cortical displacement as a function of eccentricity. Figure 4D shows a good agreement between RS- and VFM-based eccentricities (V1 ➤ V2: *R* = 0.97, *p* < 0.0001; V1 ➤ V3: *R* = 0.70, *p* < 0.0001).

**Figure 5:**
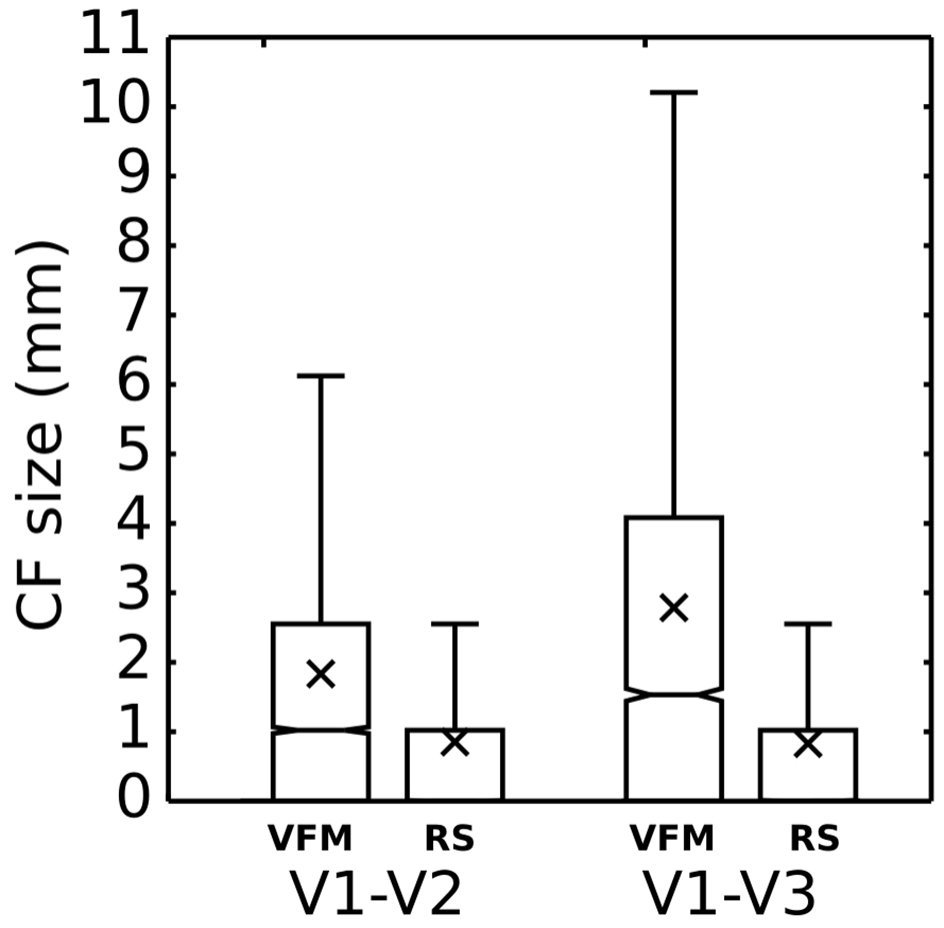
**V1-referred connective field size during visual field mapping and resting state scans grouped over participants (*N* = 4)**. Resting state based CF size is generally smaller than their visual field mapping based CF size (V1 ➤ V2: *p* < 0.0001, KS-test = 0.240; V1 ➤ V3: *p* < 0.0001, KS-test = 0.0001). CF size does not increase in the visual hierarchy when measured during resting state (*p* = 0.0065, KS-test = 0.015). A cutoff threshold of 0.35 VE was applied.

Figure 5 shows VFM- and RS-based V1-referred CF size distributions for V2 and V3 (data grouped over all scans and participants, *N* = 4). RS-based CF size tend to be smaller than those estimated based on VFM data (V1 ➤ V2: *p* < 0.0001, KS-test = 0.240; V1 ➤ V3: *p* < 0.0001, KS-test = 0.0001). Moreover, we cannot confirm a difference in RS-based CF size estimates for V1 ➤ V2 or V1 ➤ V3 (*p* = 0.0065, KS-test = 0.015).

**Figure 6:**
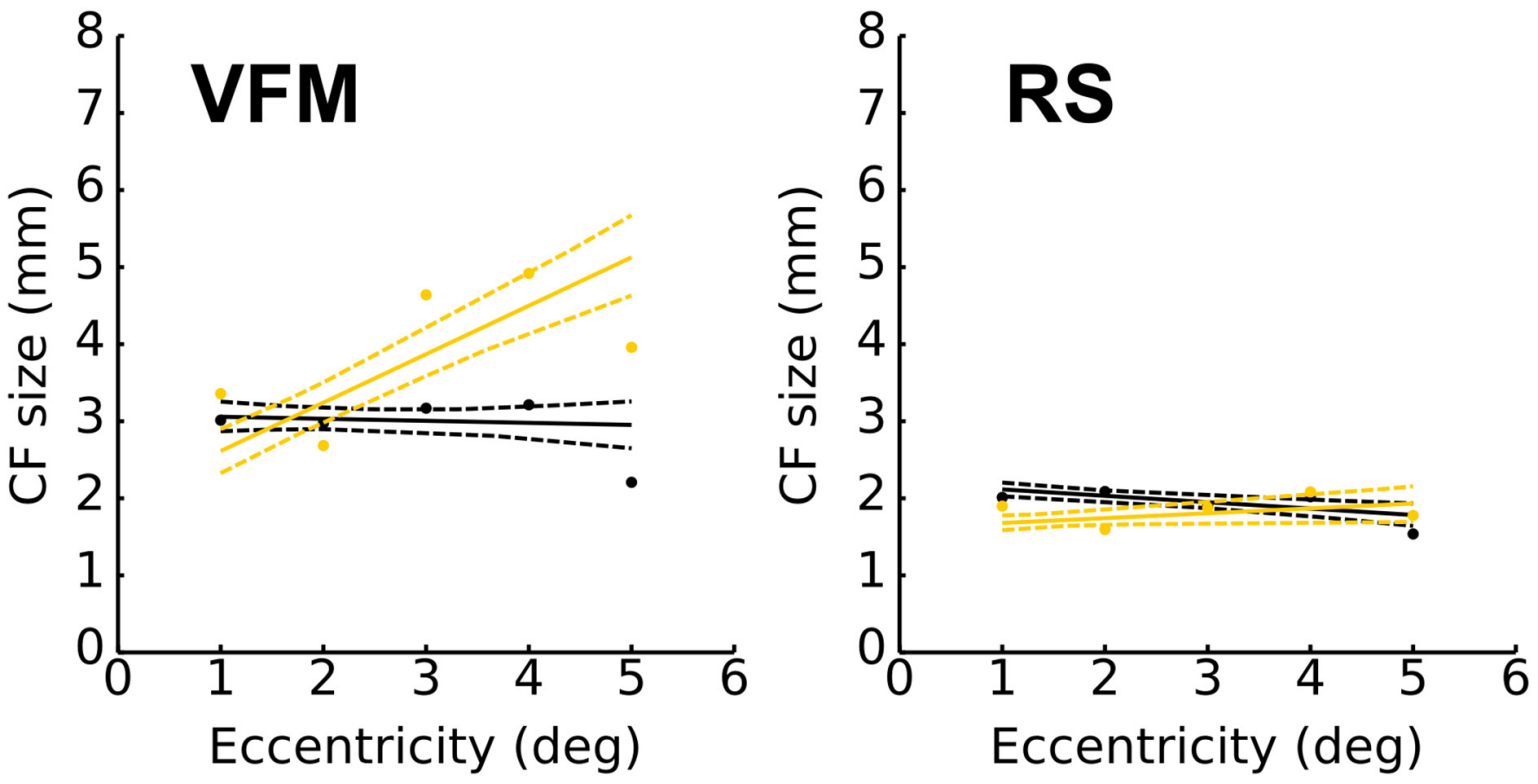
**Relation between eccentricity and V1-referred connective field size in visual areas V2 (black) and V3 (yellow) grouped over participants (*N* = 4)**. Resting state based size estimates do not increase with eccentricity. Eccentricity was binned in intervals of 1°. Dots indicate the mean of VE-weighted CF size for each bin. Linear fits were calculated for these means. Dashed lines correspond to the 95% bootstrap confidence interval of the linear fit (1000 iterations). A cutoff threshold of 0.35 VE was applied.

Figure 6 plots the relationship between CF size and eccentricity for VFM- and RS-based estimates. The left panel shows that VFM-based CF size estimates for V1 ➤ V2 do not increase significantly with eccentricity (*black line*), whereas those for V1 ➤ V3 do (*yellow line*). The right panel shows that RS-based CF size for V2 (*black line*) and V3 (*yellow line*) do not increase significantly with eccentricity.

Together, the analyses shown in Figures 5, 6 show that RS-based CF size estimates are smaller than those estimated based on VFM. In RS, CF size does not appear to increase with eccentricity, neither within the visual hierarchy.

## Discussion

### Connective field models can be estimated based on resting state data

We have shown that connective field (CF) modeling can be based on resting state (RS) data. This indicates that spontaneous blood-oxygen level dependent (BOLD) co-fluctuations in the early visual cortex state preserves fine-grained topographic connectivity structure. While this preservation of topographic connectivity corroborates results of previous studies (Heinzle et al., 2011; Raemaekers et al., 2013) our study goes beyond these by examining both the topography and the spatial properties of the functional connections. In order to assess the statistical significance of our CF estimates, we determined a variance explained (VE) cutoff threshold taking into account the VE of CF models based on surrogate RS data (Figure 1). This involves disrupting the phase correlations across recording sites in the source region of interest (ROI) in order to destroy the local structure of BOLD co-fluctuations. Furthermore, we examined the dependence of discrimination accuracy on the amount of data and found six minutes of scanning (240 volumes using a TR of 1.5 s at 7T) to be more than sufficient to achieve good discrimination (Figure 2).

### Agreement between resting state and visual field mapping based connective field parameters

Although data obtained during RS provide different information than data obtained during stimulation, a comparison of the maps estimated from RS to those estimated based on visual field mapping (VFM) reveals a fairly close agreement between the two (Figure 3). Some RS maps show patterns of visuotopic organization that agree well with their VFM reference (Figure 3, RS4, RS5). Nevertheless, we observed substantial variability in CF model parameters for different RS scans. We quantified the degree of agreement by measuring CF position scatter as the cortical displacement between RS- and VFM-based CF cortical positions and show that the median cortical displacement reflects the agreement observed in Figure 3 (data for other subjects are shown in Supplementary Materials). Besides the observed variability in visuotopic organization, CF size estimates obtained for RS scans were generally smaller than those obtained for VFM (Figure 5). Moreover, contrary to estimates based on VFM, RS-based CF size did not increase with eccentricity neither throughout the visual hierarchy (Figure 6).

### Spatial changes: possible mechanisms

In the absence of visual input, changes in CF size and variability in CF position may reflect a reduction in the amount of spatial integration and selectivity, respectively. Possible mechanisms underlying these changes in CFs may involve temporal restructuring of corticothalamic network activity in a state-dependent way (Mastronarde, 1989; Wörgötter et al., 1998; Andolina et al., 2007; Britz and Michel, 2011), as well as intracortical processing mediated by horizontal connections and feedback signals from higher cortical stages (Rao and Ballard, 1999; Steriade, 2000; Llinás and Steriade, 2006; Botelho et al., 2014; Schmid and Keliris, 2014). Decreased corticothalamic feedback and cortical lateral inhibition in the absence of visual input likely plays a role in the shrinkage of CFs, as well as in the reduced visuotopic organization observed on the higher-scatter CF maps. These input changes might adjust the balance between excitation and inhibition in cortical neuronal populations that eventually shapes cortico-cortical connectivity as a function of stimulation, behavioral context, and physiological state (Kosslyn et al., 1995; Lehmann et al., 1998; Rao and Ballard, 1999; Steriade, 2000; Martínez-Trujillo and Treue, 2004; Slotnick et al., 2005; Womelsdorf et al., 2008; Greenberg et al., 2012; Haak et al., 2012). During resting state, a variety of ongoing processes may modulate connectivity between visual areas. In particular, the transitional period from wakefulness to sleep leads to a progressive inhibition of synaptic transmission through thalamic relay neurons (Steriade, 2000; Llinás and Steriade, 2006), which is another possible cause to the changes observed.

Another reason to speculate that there may be differences between the RS and VFM results is related to the origin of the BOLD signal. Given that the majority of the brain's energy budget is devoted to ongoing intrinsic activity (i.e., RS), the metabolic costs of the adjustment between excitation and inhibition may reflect in the BOLD signal. The relative contribution of excitation and inhibition to the BOLD signal changes between the RS and VFM scans. Inhibitory functions, which may be supported more by oxidative mechanisms than by excitatory signaling, may contribute less to the measured BOLD signal (Buzsaki et al., 2007). As a consequence, resting state BOLD co-fluctuations may provide a different picture of the neural connections.

### Limitations and future directions

The current study assesses CF properties in four healthy participants. Even though the results are consistent between participants, further studies involving more participants are advised. Moreover, the CF models were estimated based on entire RS scans. As such, they only estimate average CF properties and do not capture temporal variations in these. To establish the possible neural mechanisms underlying the observed changes in CF properties, further research is still necessary. In its current implementation, the present method cannot determine the precise factors that contribute to this variability. Large-scale network interactions, physiological processes and measurement noise might all influence the variability observed. Important to note is, however, that biologically inspired methods like pRF and CF modeling that emphasize local connectivity are generally robust to global effects like physiological noise.

In future studies, extending the present analysis with dynamic functional connectivity metrics (Sakoğlu et al., 2010; Kiviniemi et al., 2011; Allen et al., 2012; Hutchison et al., 2013b), might help to disclose relevant temporal and spatial repertories in various experimental conditions allowing to study phenomena that unfold over time, such as attention, contextual modulation, and object recognition. Adding independent measures of neural activity like electroencephalography (Yuan et al., 2012) or other neurophysiological recordings seems a promising path to capture relevant temporal variations in neural activity. Future analyses could also take into account simultaneously recorded physiological data and draining veins in the preprocessing of the data, as these are known to influence resting state functional connectivity estimates (Birn et al., 2001; Logothetis et al., 2009; Winawer et al., 2010; Heinzle et al., 2011; Haak et al., 2013). Lastly, it should be noted that some of the possible mechanisms underlying changes in CF properties are based on animal models (Wörgötter et al., 1998; Steriade, 2000; Haupt et al., 2004; Llinás and Steriade, 2006; Andolina et al., 2007; Womelsdorf et al., 2008). Because certain experimental manipulations are not possible in human subjects, comparative approaches between humans and animal models are needed to bridge the gap in RS-fMRI investigations (Hutchison and Everling, 2012; Mantini et al., 2012). Examining the correspondence of functional and anatomical connectivity in homologous brain architectures will help to further elucidate the mechanisms underlying neural activity.

## Concluding remarks

We have shown that CF estimates can be obtained based on RS data. We observed good agreement can be observed between RS- and VFM-based maps, and between different RS-based maps. This implies that local functional connectivity in visual cortical areas during resting state, as measured with CF modeling, may reflect the underlying neural architecture. However, we found that CF estimates may vary between RS scans even for high VE scans. The present study cannot determine to what extent this variability is explained by genuine changes in the neural properties of the visual system or by various external sources of noise. Nevertheless, we show that neural properties such as CF maps and CF size can be derived from RS data.

### Conflict of interest statement

The Review Editor Jonathan Winawer declares that, despite having collaborated with authors Koen V. Haak, Ben Harvey, Serge O. Dumoulin, Remco Renken, and Frans W. Cornelissen two years ago, the review process was handled objectively and no conflict of interest exists. The authors declare that the research was conducted in the absence of any commercial or financial relationships that could be construed as a potential conflict of interest.
