# Supplementary material for: Cortical connective field estimates from resting state fMRI activity
Source: Front Neurosci. 2014 Oct 31;8:339. doi: 10.3389/fnins.2014.00339 (PMC4215614; doi:10.3389/fnins.2014.00339)
Supplement: Supplementary file 1 [file DataSheet1.PDF]

SUPPLEMENTARY MATERIALS

Table of content:

1. *Visualization of connective field parameters.* *1*
   Subject 1 1
   Subject 2 2
   Subject 4 3
2. *Relation between eccentricity and V1-referred connective field size.* *4*

1. Visualization of connective field parameters.

Subject 1

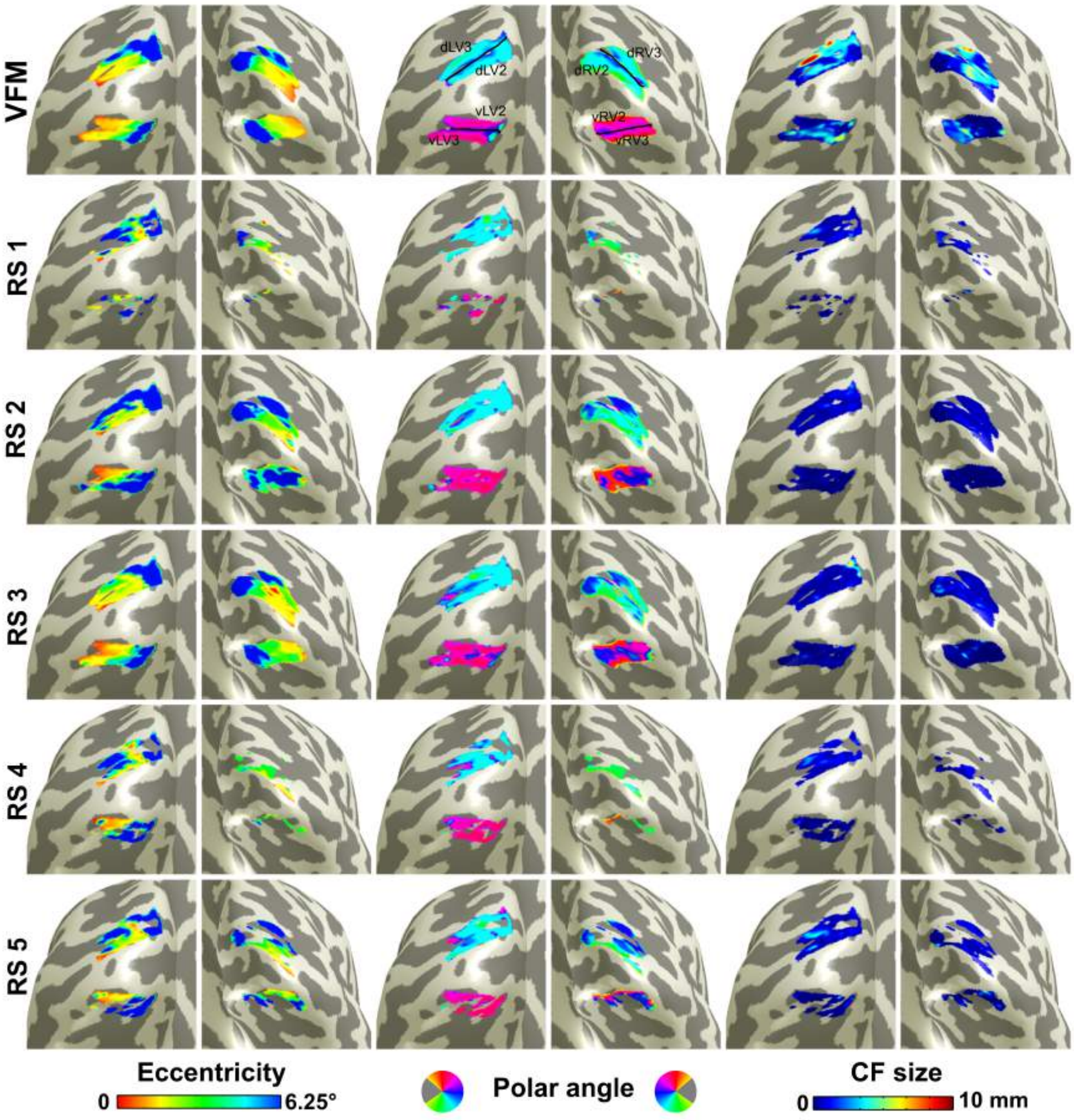

Positional displacement in CF cortical location (in mm) for RS1 to RS5 is:
V1➤V2: Median (MAD) = 5.1 (6.9); 12.5 (8.3); 10.6 (8.3); 5.2 (6.9); 7.1 (5.9). Total: 8.3 (7.3).
V1➤V3: Median (MAD) = 13.3 (5.7); 12.1 (4.2); 8.2 (5.0); 13.5 (6.0); 13.8 (5.0). Total: 11.3 (4.9).

Subject 2

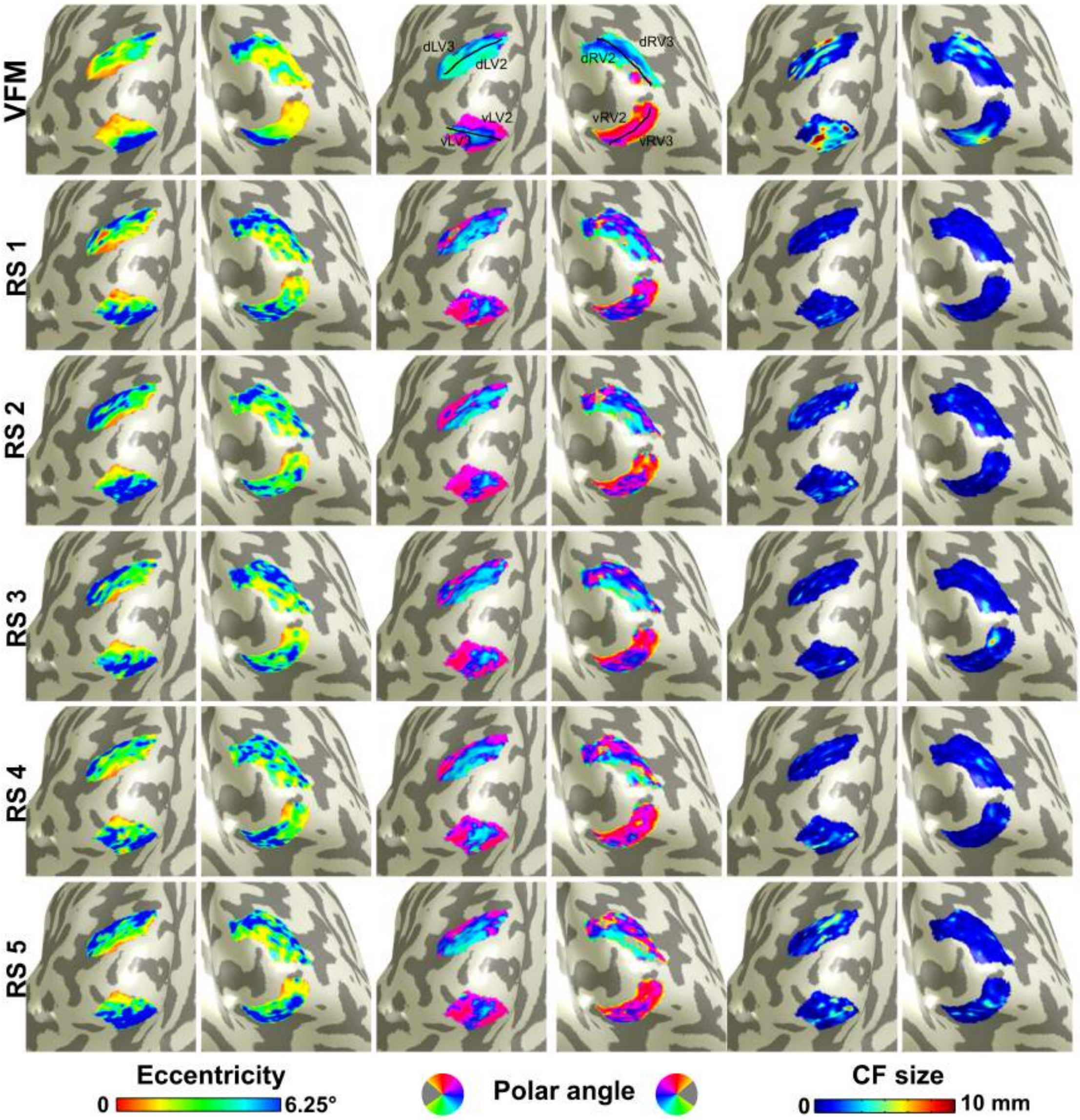

Positional displacement in CF cortical location (in mm) for RS1 to RS5 is:

V1➤V2: Median (MAD) = 9.1 (8.8); 15.5 (9.7); 12.4 (9.7); 8.2 (8.8); 10.6 (5.4). Total: 11.2 (8.5).

V1➤V3: Median (MAD) = 12.0 (5.9); 12.6 (6); 12.7 (5.1); 13 (5.8); 14 (5.6). Total: 13.1 (5.6).

Subject 4

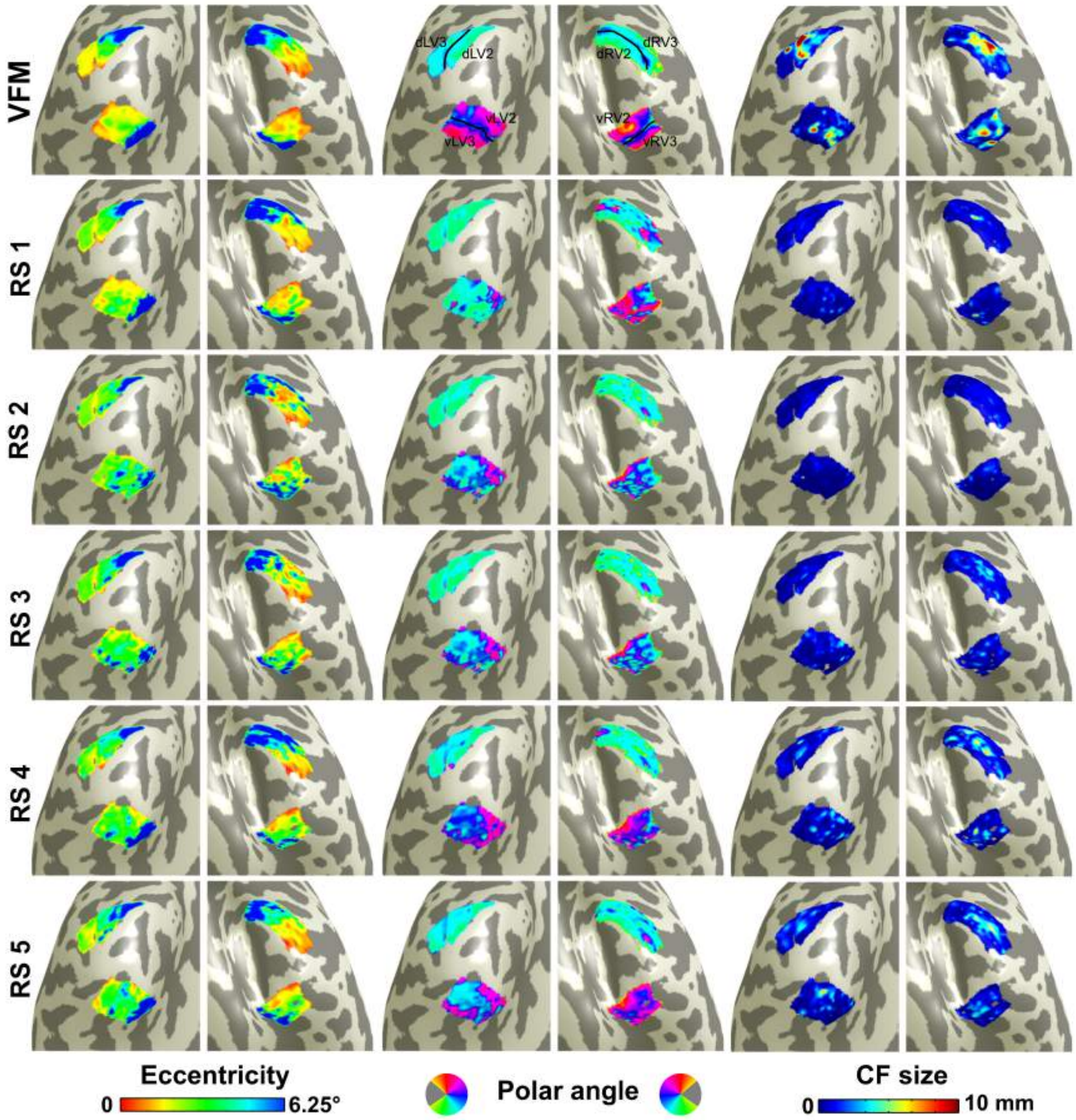

Positional displacement in CF cortical location (in mm) for RS1 to RS5 is:
V1➤V2: Median (MAD) = 6.1 (4.2); 10.3 (6.6); 8.1 (4.9); 5.6 (3.9); 4.8 (3.9). Total: 6.6 (4.5).
V1➤V3: Median (MAD) = 8.5 (5.0); 13.6 (6.4); 10.2 (5.0); 7.9 (4.5); 7.5 (4.3). Total: 9.1 (5.0).

2. Relation between eccentricity and V1-referred connective field size.

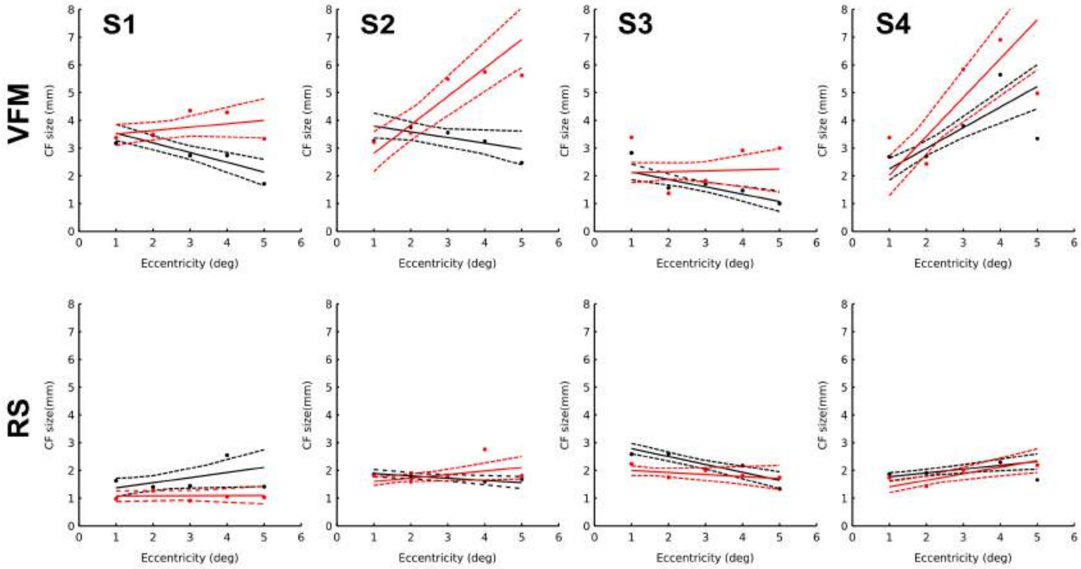

Relation between eccentricity and V1-referred connective field size in visual areas V2 (*red*) and V3 (*black*). Eccentricity was binned in intervals of 1 deg. Color dots indicate the mean of EV weighted CF size for each bin. Linear fits were calculated for these means. Dashed lines correspond to the 95% bootstrap confidence interval of the linear fit (1000 iterations). A cutoff threshold of 0.35 EV was applied.
